# Genistein protects against hyperglycemia and fatty liver disease in diet-induced prediabetes mice *via* activating hepatic insulin signaling pathway

**DOI:** 10.3389/fnut.2022.1072044

**Published:** 2022-12-08

**Authors:** Nana Zhang, Weiyue Zhang, Xinxin Guo, Jianlin Liu, Shuying Li, Hongtai Zhang, Bei Fan

**Affiliations:** Key Laboratory of Agro-Products Processing, Ministry of Agriculture, Institute of Food Science and Technology, Chinese Academy of Agricultural Sciences, Beijing, China

**Keywords:** genistein, high-fat diet, hyperglycemia, insulin resistance, fatty liver

## Abstract

**Introduction:**

Insulin signaling *via* the insulin receptor (IR) may be associated with the amelioration of diet-induced metabolic syndrome. Genistein, a soy isoflavone, has been suggested to play a role in the amelioration of high-fat diet-induced metabolic disorders.

**Methods:**

Here, we aimed to explore whether genistein regulates glucose and hepatic lipid by activating the insulin signaling pathway in diet-induced obesity mice.

**Results:**

We showed that treatment of western-style diet-fed mice with genistein (60 mg/kg) significantly improved insulin resistance with decreased hyperglycemia and HOMA-IR index. These effects were linked to activating hepatic IRβ/PI3K/Akt signaling. Furthermore, genistein suppressed gluconeogenesis and promoted glycogen synthesis to maintain glucose homeostasis by increasing the phosphorylation of hepatic FOXO1/GSK3β *in vivo* and *in vitro*. The reduced level of insulin and upregulation of insulin signaling in genistein-treated mice also lead to an increase in hepatic energy status by inducing energy-sensing AMPK, reducing hepatic SREBP1c/ACC/FAS without affecting β-oxidation to prevent hepatic lipid accumulation. The protective effect of genistein on hepatic lipid accumulation was also validated *in vitro*. Besides, genistein had little effect on improvements in intestinal function and liver inflammation.

**Conclusion:**

Taken together, our results showed that genistein prevents insulin resistance and hyperglycemia through improvements in hepatic function. This study provides new insight into the mechanisms of genistein mediating glucose metabolism and suggests that genistein may be a promising diet ingredient for preventing prediabetes and hepatic lipid accumulation.

## Introduction

The global prevalence of type 2 diabetes is extremely serious and prediction indicates that diabetes will affect 643 million in 2030 (1). Prediabetes is viewed as an increased risk for diabetes (2, 3) and people with prediabetes have an increased risk for cardiovascular disease, renal disease, and mortality (4). More than 70% of prediabetes will develop into diabetes without proper intervention (5). Prediabetes represents the ideal stage that can be targeted to prevent type 2 diabetes (6). The progression of prediabetes to diabetes can be prevented by lifestyle changes as well as some alternative therapies, such as natural products and functional foods (7). Thus, identifying safe and efficient natural active products targeting glucose homeostasis provides an appealing option for early intervention of prediabetes.

Prediabetes is coupled with simultaneous development of insulin resistance (8). The liver, an insulin-sensitive organ, plays a central role in regulating glucose and lipid homeostasis. Insulin function is regulated by activating the insulin receptor (IR)/phosphatidylinositol 3kinase (PI3K)/AKT signaling pathway (9). In response to metabolic disorders, hepatic insulin resistance is one of the early developed pathologies. Insulin resistance in liver has major consequences on the abnormalities observed in the metabolic syndrome (10). Insulin resistance is recognized to stimulate hepatic excessive lipid storage, which can aggravate insulin resistance (11). It has been demonstrated that improving insulin resistance is of great potential for treating hyperglycemia and lipid storage in liver.

Genistein [5,7-dihydroxy-3-(4-hydroxyphenyl)chromen-4-one] is a naturally occurring isoflavone that can be derived from legumes such as *Glycine max* (L.) Merr. (soybeans), *Vicia faba* L. (fava bean), and *Pueraria lobata* (Willd.) Ohwi (kudzu) (12). Studies have shown that genistein has therapeutic effects on depression, neurodegeneration (13), bone health (14), and postmenopausal breast cancer (15). Recent and accumulating evidence has revealed that genistein reduces lipid accumulation in liver and alleviates insulin resistance and hyperglycemia (16–18). However, the exact mechanisms by which genistein regulates glucose metabolism in the liver have not been fully elucidated.

In this study, we sought to investigate the impact of the administration of genistein on insulin resistance in high-fat-fed mice. We also aimed at analyzing the role of insulin signaling in mediating the effects of genistein on glucose metabolism and hepatic lipid accumulation. We reported that genistein could prevent prediabetes by improving hepatic function and also suggest that the activation of hepatic insulin signaling contributes to the beneficial effects.

## Materials and methods

### Materials

Antibodies to GAPDH, HSP90, fatty acid synthase (FAS), acetyl-CoA carboxylase (ACC), insulin receptor β (IRβ), phospho-PI3 Kinase p85 (Try458)/p55 (Try199), PI3 Kinase, Phospho-Akt (Ser473), Akt, forkhead box O1 (Foxo1), Foxo1 (pS256), glycogen synthase kinase 3 beta (GSK3β), and GSK3β (pS9), Phospho-AMPKα (Thr172), AMPKα, SREBP1 and IRβ (pY1162/1163), and HRP-conjugated secondary antibody were obtained from Cell Signaling Technology (MA, USA). IRβ (pY1162/1163) was obtained from Santa Cruz (CA, USA). Antibody to SREBP1 was from Abcam (Cambridge, UK). Protease inhibitor mixture was obtained from Roche (Basle, Switzerland). Genistein was obtained from Shenggong Biological Engineering Co., Ltd. (Shanghai, China) and the purity of genistein exceeded 98.0%. Trizol reagents were from Invitrogen (California, USA). GoScript reverse transcription (RT) system and Power SYBR Green PCR master mix were from Promega (Wisconsin, USA). The ELISA kits and bicinchoninic acid (BCA) protein assay kits were purchased from Thermo Fisher (MA, USA). Immobilon Western chemilum HRP substrate and polyvinylidene difluoride membrane (PVDF) and enhanced chemiluminescence (ECL) kits were purchased from Millipore (Darmstadt, Germany).

### Animals and treatment

Male C57bl/6J mice aged 6 weeks were from the Vital River Laboratory Animal Technology (Beijing, China). Mice were maintained in 12:12-h dark–light cycle with free access to food and water. After acclimatized for 2 weeks, mice were randomly assigned to three groups (*n* = 12). The groups were fed either a chow diet, high-fat diet (HFD) diet, or HFD + genistein (60 mg/kg), which are identified as *Chow*, *HFD*, and *Genistein*, respectively. Chow group and HFD group were administrated with 100 μl distilled water in the absence or presence of 60 mg/kg genistein. Treatment started concomitantly with the introduction of HFD diet. The animal studies were approved by the Animal Care and Use Committee of the School of Basic Medical Sciences, Inner Mongolia Medical University. The animal care and treatment protocols followed the National Institutes of Health guidelines (GB 14925-2010).

### Cell culture and treatment

HepG2 cells (ATCC; Maryland, USA) were cultured in Dulbecco’s Modified Eagle’s Medium (DMEM) amended with 10% fetal bovine serum (FBS) (Invitrogen, CA, USA) and 1% antibiotics at 37°C in 5% CO_2_. To establish a fatty HepG2 cell model, HepG2 was induced by treating with 1mM FFA mixture (oleic acid:palmitic acid = 0.66 mM:0.33 mM) (Solarbio Co., Ltd., Beijing, China) for 24 h. The differentiation of HepG2 cells was confirmed by measuring triglyceride levels. Primary hepatocytes were isolated as previously reported (19). Briefly, primary hepatocytes were isolated from male C57BL/6 mice with HBSS (Hyclone, UT, USA) and collagenase IV (Gibco, NY, USA), seeded in a six-well plate in William’s E medium for 24 h. The cells were treated with genistein for 24 h after starved for 4 h.

### Histological analysis

Livers were fixed in 4% formalin, paraffin embedded, and then sectioned. The sectioned liver tissues were stained with hematoxylin and eosin (HE). Morphological changes were observed under microscope (Carl Zeiss AG, Jena, Germany).

### Inflammatory cytokine analysis

Cytokine levels in the serum and colon were determined using ProcartaPlex Mouse Cytokine panel following the instructions from the manufacturer and performed with a Luminex MAGPIX System (Luminex, TX, USA).

### Reverse transcription-qPCR

Total RNA was extracted from liver using Trizol. Complementary DNA (cDNA) was synthesized from total RNA with PrimeScript reverse transcriptase and random primers. Gene expression was measured on a real-time PCR system (ABI 7500) using Power SYBR Green PCR master mix (Applied Biosystems, Carlsbad, CA) and. Relative quantification was calculated using the ΔΔCt method with HFD-treated mice as the group of reference. The primer sequences are listed in Table 1.

**TABLE 1:** List of primers used in this study.

Genes	Gene bank accession	Primers	Primers sequence	Amplicon size (bp)
*G6pc*	NM_008061.4	Forward	5′- GTTTCCCTTGACAACGCGAG -3′	181
		Reverse	5′- GTAACACTCCGGTCTCCACA -3′	
*Pck1*	NM_011044.3	Forward	5′- ACTTTCCGGCGTGGTACATA -3′	92
		Reverse	5′- CCCGCTCAGACAGTCAAGTT -3′	
*Gk*	NM_008194.3	Forward	5′- GTGGAAACTCCGACTGGAGA -3′	70
		Reverse	5′- AGTGGGATCTGGAATTGCCCT -3′	
*Pfkl*	NM_008826.5	Forward	5′- TAGGGTAACGTCTGGCAACA -3′	182
		Reverse	5′- GATTCCCGAGTCCAGAGGGA -3′	
*Chrebp*	NM_001359237.1	Forward	5′- TCACAGATGCGAAGGACGTG -3′	77
		Reverse	5′- GATGTCTCTTCCCCTGCCCT -3′	
*Pklr*	XM_006501135.4	Forward	5′- GGCTCTATGCGTGACCTCAG -3′	137
		Reverse	5′- CACCATCAGGTGGGTGTGAC -3′	
*Glut2*	NM_031197.2	Forward	5′- ACTCAAGGAAGGTCAAGCCG -3′	110
		Reverse	5′- TCGAAAGGCCAGTAGGTCAC -3′	
*Glut4*	NM_001359114.1	Forward	5′- GTAAACCCCGGGATCCAACA -3′	88
		Reverse	5′- AAGCCCAAATCGTGGGAAGG -3′	
*Cd36*	NM_001159558.1	Forward	5′- ATCATCTTGGCCCGGTGCAT -3′	154
		Reverse	5′- GTCGGTCCTGACGTGGTTAT -3′	
*Fabp4*	NM_024406.3	Forward	5′- GCTACTTTAGTGGCGTCTGC -3′	131
		Reverse	5′- GGTCGAACAGTGGTAGAGCA -3′	
*Scd1*	NM_009127.4	Forward	5′- AAGAGTCTTTGTGTGCGGCT -3′	143
		Reverse	5′- TCGAAGAGCCGAAAGTCCAG -3′	
*Pgc1a*	NM_001402988.1	Forward	5′- CCGTGGACTTGTCTTGCTTG -3′	194
		Reverse	5- CATGGCCTCCGACTGTTGTC -3′	
*Ppar*α	XM_011245517.4	Forward	5′- TCAGCTCCTACATCGGGTCA -3′	156
		Reverse	5′- ATTTGTCCCAGAGTGTGCCG -3′	
*Cpt1*	NM_011517.2	Forward	5′- TCGGCGACTCGTTTGTAGAT -3′	84
		Reverse	5′- CGACTAGTTGGTTTCCACCG -3′	
*Il-1*β	XM_006498795.5	Forward	5′- CAGCGAGTCCCAGTGTTCTT -3′	155
		Reverse	5′- CACGACGGATTACAGGGGAA -3′	
*Il-6*	NM_001314054.1	Forward	5- GTGAAGTGTTCAGCCTCCGA -3′	113
		Reverse	5′- GACGTTCACGTAGTAGCAACA -3′	
*Tnf*α	NM_001278601.1	Forward	5′- TCGGCTACCCAACATGGAAC -3′	99
		Reverse	5′- TATCGTTTAGCCGACTGCCA -3′	
*Tlr4*	NM_021297.3	Forward	5′- GGTACGTAAACCGGAATCGG -3′	74
		Reverse	5′- TCTCGTGACTTGGAGGAACG -3′	

### Western blot

Western blot was performed based on protocol published before (20). The protein lysates of liver were separated on sodium dodecyl sulfate-polyacrylamide gel electrophoresis and transferred to a polyvinylidene difluoride membrane. The membrane was immunoblotted with primary antibodies followed by incubation with horseradish peroxidase-coupled secondary antibodies.

### Data analysis

Data were presented as means ± SEM. Two-tailed Student’s *t*-test or One-way analysis of variance (ANOVA) were used to evaluate the data with GraphPad Prism 7.0 program (San Diego, USA). The data were considered statistically significant at *P* < 0.05.

## Results

### Genistein treatment prevents hyperglycemia, glucose intolerance, and insulin resistance in diet-induced obese mice

**FIGURE 1:**
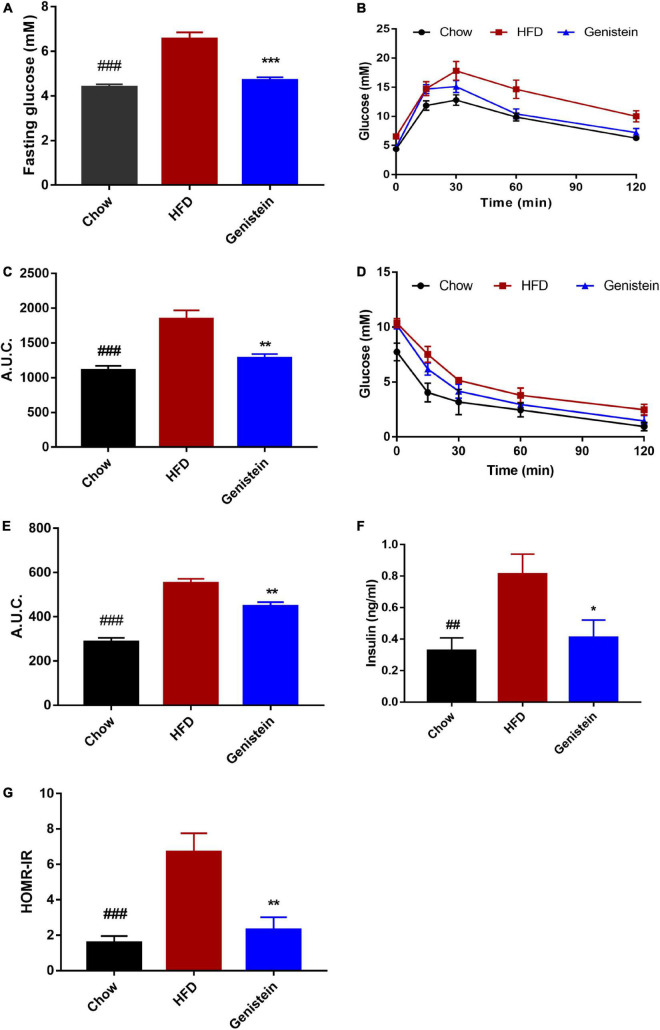
Genistein treatment prevents hyperglycemia, glucose intolerance, and insulin resistance in diet-induced obese mice. **(A)** Fasting blood glucose levels. **(B)** Mice were fasted overnight and subjected to oral glucose tolerance tests (OGTT). **(C)** Area under the curve in OGTT. **(D)** Mice were fasted for 4 h and were carried out insulin tolerance tests (ITT). **(E)** Area under the curve in ITT. **(F)** Fasting insulin levels. **(G)** The homeostatic model assessment of insulin resistance (HOMA–IR) index. Data are presented as means ± SEM; *n* = 6 mice/group. ^##^*p* < 0.01 and ^###^*p* < 0.001 for Chow vs. HFD. *p < 0.05, **p < 0.01 and ***p < 0.001 for Genistein vs. HFD.

To investigate the impact of genistein on glucose metabolism, we made use of the HFD-induced prediabetics mice. Fasting blood glucose level was significantly elevated by high-fat diet while genistein treatment prevented fasting hyperglycemia (Figure 1A). Oral glucose tolerance tests (OGTT) were conducted at 12 weeks post genistein treatment. As expected, HFD reduced glucose tolerance compared with chow diet (Figures 1B,C). However, genistein enhanced glucose clearance, as indicated by a decrease in AUC of OGTT (Figure 1C). Insulin sensitivity was directly identified by i.p. insulin tolerance test (ITT) at 12 weeks. The test revealed that genistein-treated mice had elevated insulin sensitivity compared with the HFD group (Figures 1D,E). Consistent with improved glucose tolerance, genistein mice had lower fasting serum insulin and the homeostatic model assessment of insulin resistance (HOMA-IR) index, compared to HFD-fed mice (Figures 1F,G). Taken together, our findings suggested that genistein had a preventive effect on HFD-induced hyperglycemia and insulin resistance.

### Genistein improves insulin sensitivity through activating hepatic insulin receptor β- phosphatidylinositol 3kinase-AKT pathway

**FIGURE 2:**
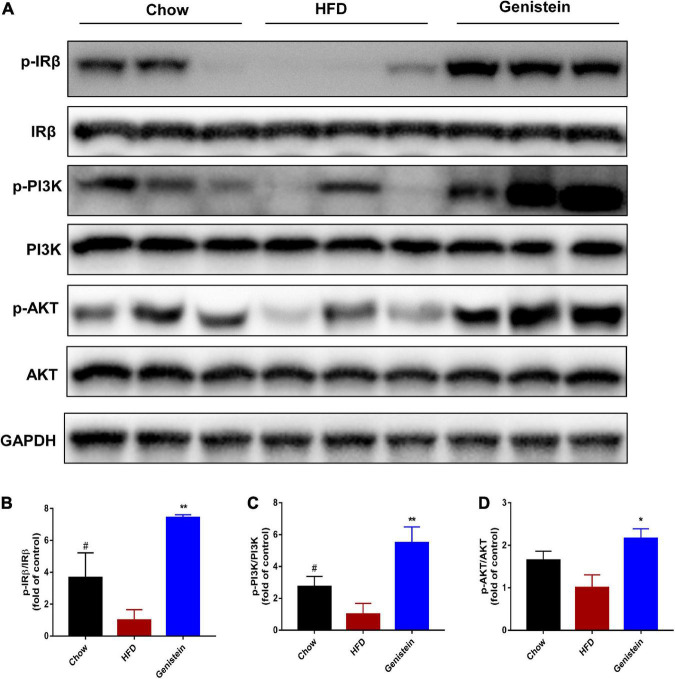
Genistein improves insulin sensitivity through activating hepatic IRβ/PI3K/AKT pathway. **(A)** Western blot for p-IRβ, IRβ, p-PI3K, PI3K, p-AKT and AKT in mice liver. **(B)** Quantification for the phosphorylation of IRβ. **(C)** Quantification for the phosphorylation of PI3K. **(D)** Quantification for the phosphorylation of AKT. HFD-fed mice were administered daily with genistein by oral gavage for 12 weeks. Data are presented as means ± SEM; *n* = 4–5 mice/group. ^#^*p* < 0.05 for Chow vs. HFD. *p < 0.05 and **p < 0.01 for Genistein vs. HFD.

Since IR and its downstream signaling molecules have been shown to be crucial in medicating insulin function and regulating hepatic glucose metabolism, we sought to evaluate whether hepatic IRβ/phosphatidylinositol 3-kinase (PI3K)/AKT pathway could be linked to the improved insulin sensitivity seen in genistein-treated mice. We used western blotting to assess the protein levels of insulin signaling molecules in the liver sample of HFD and genistein receivers. The phosphorylation of IRβ was upregulated in the liver of genistein mice (Figures 2A,B). Genistein mice showed a tendency of higher PI3K phosphorylation in liver as compared with vehicle-treated HFD-fed mice (Figures 2A,C). Our findings also revealed that 12 weeks of HFD feeding significantly suppressed the insulin sensitivity, showing an impaired AKT phosphorylation in the liver (Figures 2A,D). We found genistein treatment induced a substantial increase in AKT phosphorylation in the livers, suggesting an improved ability to induce hepatic AKT activation in genistein-treated mice (Figures 2A,D). Overall, genistein mitigated insulin resistance *via* activating the hepatic IRβ-PI3K-AKT pathway.

### Genistein lowers hyperglycemia through inhibiting hepatic glucose production *in vivo* and *in vitro*

**FIGURE 3:**
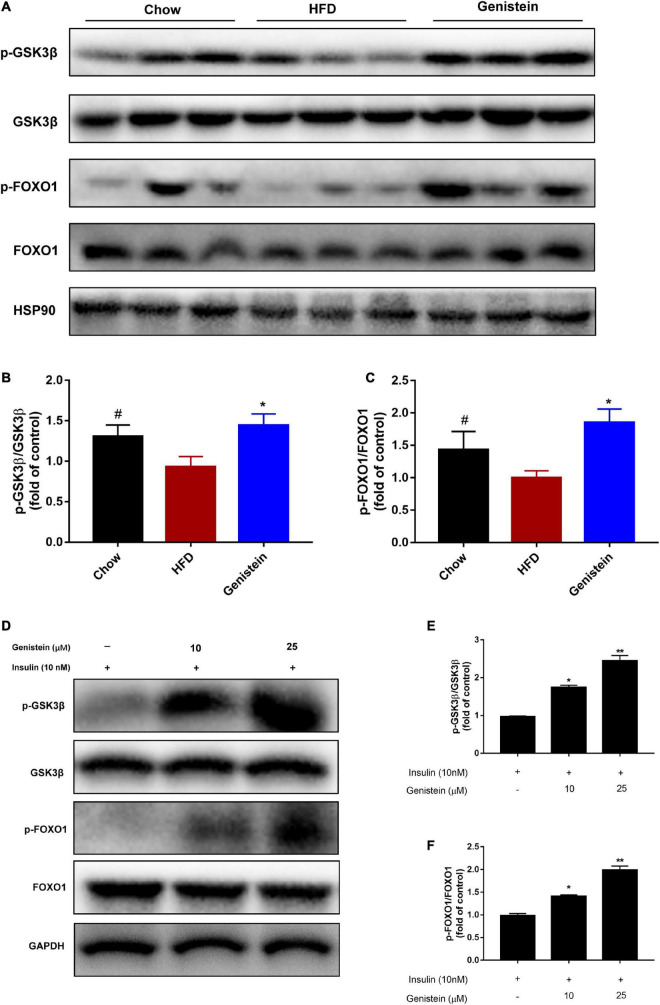
Genistein lowers hyperglycemia through inhibiting hepatic glucose production *in vivo* and *in vitro* **(A)** Western blot for p-GSK3β, GSK3β, p-FOXO1, FOXO1 in mice liver. **(B)** Quantification for the phosphorylation of GSK3β. **(C)** Quantification for the phosphorylation of FOXO1. Data are presented as means ± SEM; *n* = 4–5 mice/group. ^#^*p* < 0.05 for Chow vs. HFD. *p < 0.05 for Genistein vs. HFD. **(D)** Genistein promotes the phosphorylation of GSK3β and FOXO1 in primary cultured hepatocytes. Serum-starved primary cultured hepatocytes received either no pretreatment or pretreatment with 10 or 25 μM for 24 h followed by treating 10 nM insulin. **(E)** Quantification of the phosphorylation of GSK3β. **(F)** Quantification for the phosphorylation of FOXO1. Data are presented as means ± SEM; *n* = 3. *p < 0.05 and **p < 0.01.

As genistein induced hepatic AKT phosphorylation, we next turned our attention to the liver and assessed hepatic glycometabolic pathways involved in glycogen synthesis and gluconeogenesis. Previous studies have reported that glycogen synthase kinase 3β (GSK3β) and forkhead box O1 (FOXO1) are the most important downstream factors of AKT involved in regulating glucose production. Consistent with these data, compared with the HFD group, genistein treatment group increased the level of phosphorylated GSK3β and FOXO1 in the liver (Figures 3A–C), indicating that inhibiting glycogen synthesis and gluconeogenesis contributed to the glucose homeostasis in genistein group. Primary cultured hepatocytes were also used to evaluate the effect of genistein on glycogen synthesis. As shown in Figures 3D–F, genistein increased the phosphorylation of GSK3β and FOXO1 induced by insulin in a dose-dependent manner. Collectively, these findings demonstrated that genistein-mice displayed decreased glycogen synthesis and gluconeogenesis in the liver.

### Genistein regulates the expression of glucose metabolism genes

**FIGURE 4:**
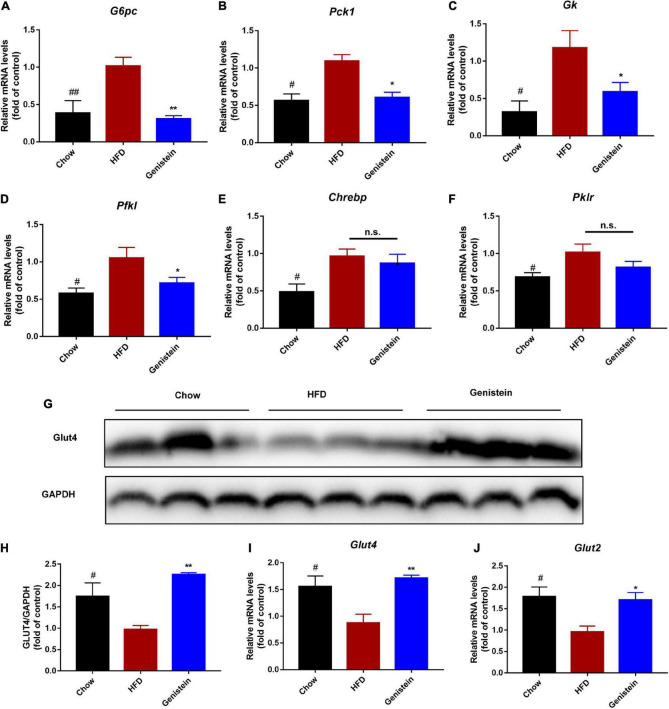
Genistein regulates the expression of glucose metabolism genes **(A–F)** qRT-PCR analysis of *G6pc*, *Pck1*, *Gk*, *Pfkl*, *Chrebp*, and *Pklr* in liver tissue of different groups. **(G)** Western blot for Glut4 in mice liver. **(H)** Quantification for Glut 4. **(I,J)** qRT-PCR analysis of *Glut4* and *Glut2* in liver tissue of different groups. Data are presented as means ± SEM; *n* = 4–5 mice/group. ^#^*p* < 0.05 and ^##^*p* < 0.01 for Chow vs. HFD. *p < 0.05 and **p < 0.05 for Genistein vs. HFD.

To further understand what might affect the glucose homeostasis, we sought to evaluate whether genistein regulates the expression of glucose regulatory genes. Gene expression studies showed that glucose-6-phosphatase (*G6pc*), a key enzyme in gluconeogenesis, was reduced in genistein-treated mice (Figure 4A). In agreement, other gluconeogenic genes, phosphoenolpyruvate carboxy kinase 1 (*Pck1)*, glucokinase (*Gk*) and phosphofrutokinase (*Pfkl*) were also significantly decreased in genistein groups although mRNA expression of carbohydrate response element binding protein (*Chrebp*) and phosphofrutokinase (*Pfkl*) was not greatly altered in the liver as compared with HFD-fed mice (Figures 4B–F), suggesting reduced availability of glucose as substrate and glycolysis. Concordant with lower glucose metabolism, genistein upregulated the protein level of glucose transporter 4 (GLUT 4) in liver tissues (Figures 4G,H). Besides, the mRNA levels of hepatic glucose transporter, *Glut2* and *Glut4* were increased in the liver of genistein treated versus vehicle-treated HFD-fed mice (Figures 4I,J). In summary, these data supported that genistein regulated glucose metabolism in the liver.

### Genistein prevents fatty liver induced by high-fat diet

**FIGURE 5:**
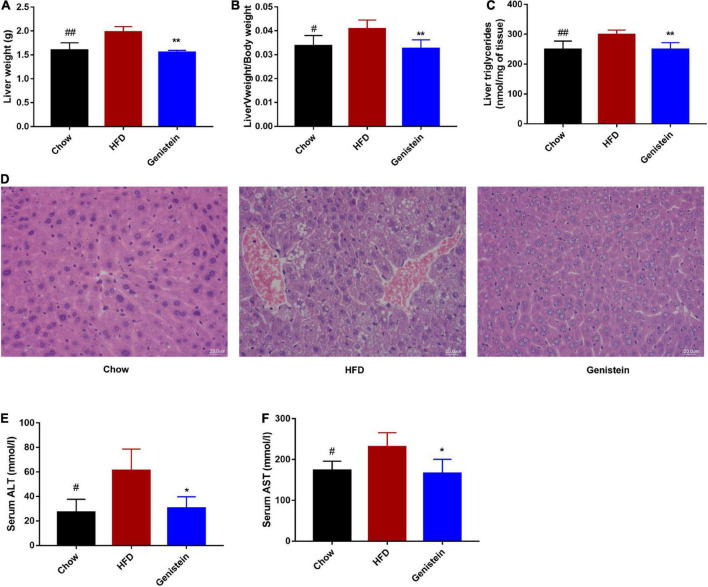
Genistein prevents fatty liver induced by HFD **(A)** Live tissues were harvested and weighted during necropsies. **(B)** The ratio of liver weight to body weight. **(C)** The content of triglycerides in liver tissues. **(D)** Representative images of liver H&E staining of liver tissues. Scale bars, 20 μm. **(E,F)** Plasma ALT and AST levels. Data are presented as means ± SEM; *n* = 4–5 mice/group. ^#^*p* < 0.05 and ^##^*p* < 0.01 for Chow vs. HFD. *p < 0.05 and **p < 0.05 for Genistein vs. HFD.

As genistein-treatment mice showed improved glucose metabolism, we next assessed whether genistein affects lipid metabolism in the liver. Liver weight, as well as the ratio of liver weight to body weight, was significantly reduced in genistein-treated mice (Figures 5A,B). Higher level of hepatic triglyceride was also noticed in HFD mice as compared to chow mice whereas genistein prevented the HFD-driven elevation in hepatic triglyceride content (Figure 5C). Consistently, HFD-induced hepatic lipid vacuole accumulation was prevented by genistein treatment, as indicated by hematoxylin and eosin (H&E) staining (Figure 5D). Additionally, genistein also prevented live injury induced by HFD, as evidenced by decreased levels of ALT and AST in the serum of genistein-treated mice (Figures 5E,F). These results suggested that genistein ameliorated fatty liver induced by HFD and may affect liver to maintain glucose homeostasis and insulin sensitivity.

### Genistein suppresses hepatic *de novo* lipogenesis

**FIGURE 6:**
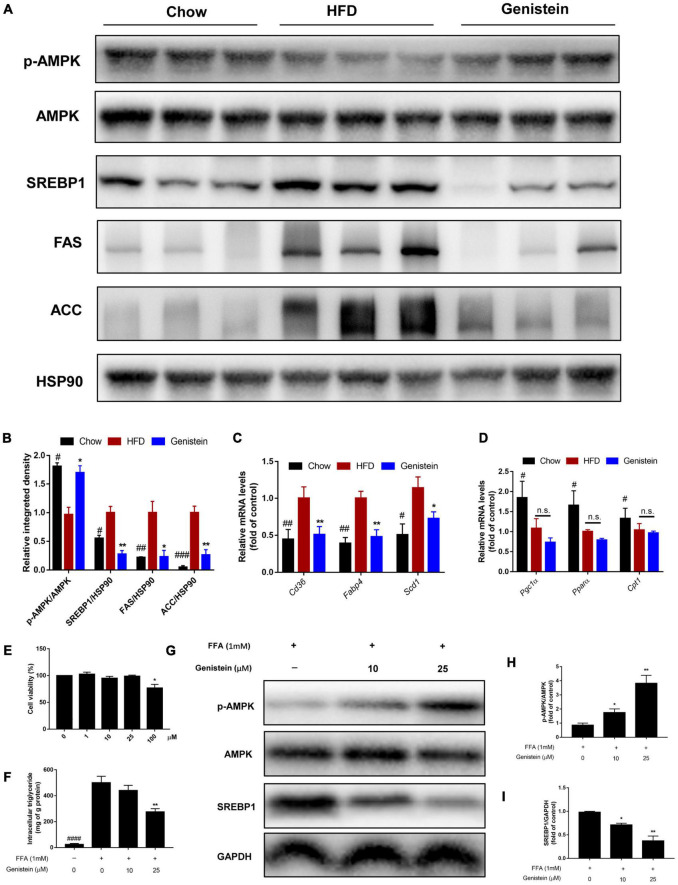
Genistein suppresses hepatic *de novo* lipogenesis **(A)** Representative western blot of p-AMPK, AMPK, SREBP1, FAS, and ACC in liver tissue. **(B)** Relative integrated density of indicated proteins in liver of mice. **(C)** The mRNA levels of *Cd36*, *Fabp4*, and *Scd1* in liver. **(D)** The mRNA levels of *Pgc1*α, *Ppar*α and *Cpt1* in liver. Data are presented as means ± SEM; *n* = 4–5 mice/group. ^#^*p* < 0.05, ^##^*p* < 0.01, and ^###^*p* < 0.001 for Chow vs. HFD. *p < 0.05 and **p < 0.05 for Genistein vs. HFD. **(E)** Cell viability was measured in HepG2 cells. **(F)** Intracellular triglyceride content in FFA-induced HepG2 cells treated with or without genistein. **(G)** Western blot for p-AMPK, AMPK, SREBP1 in FFA-induced HepG2 cells. **(H)** Quantification of the phosphorylation of AMPK. **(I)** Quantification of SREBP1 normalized to GAPDH. Data are presented as means ± SEM; *n* = 3. *p < 0.05 and **p < 0.01.

Western blotting analysis revealed that 12 weeks of HFD feeding drastically decreased the level of phosphorylation of AMPKα (Figures 6A,B). However, genistein treatment prevented the diet-induced decrease of the AMPKα phosphorylation (Figures 6A,B). To understand the underlying mechanism responsible for fatty acid metabolism in the liver of genistein-treated mice, we explored hepatic lipid metabolism pathways, including fatty acid uptake, fatty-acid β-oxidation, and *de novo* lipogenesis. We also found reduced protein expression of sterol regulatory element binding protein 1 (SREBP1), acetyl-coA carboxylase (ACC), and fatty acid synthase (FAS) in the liver of genistein-treated mice (Figures 6A,B). We revealed that the expression of cluster of differentiation 36 (*Cd36*), fatty acid binding protein 4 (*Fabp4*), and stearoyl-CoA desaturase 1 (*Scd1*) was obviously dampened by genistein treatment (Figure 6C). Unexpectedly, the mRNA levels of peroxisome proliferator-activated receptor gamma co-activator-1 (Pgc1α) carnitine palmitoyltransferase (*Cpt1a*) and *Ppar*α, which are responsible for fatty acid oxidation, were not affected by genistein (Figure 6D). We also verified its effect on hepatic lipid accumulation in HepG2 cells. Genistein didn’t affect the cell viability at concentration ≤ 25 μM (Figure 6E). Genistein inhibited the lipid formation induced by the free fatty acid (FFA) mixture (Figure 6F), by activating the AMPK/SREBP1 pathway (Figures 6G–I). These results suggested that genistein may ameliorate liver steatosis by inhibiting fatty acid synthesis and fatty acid uptake but not through enhancing lipid degradation.

### Genistein has limited effects on intestinal permeability and markers of liver inflammation

**FIGURE 7:**
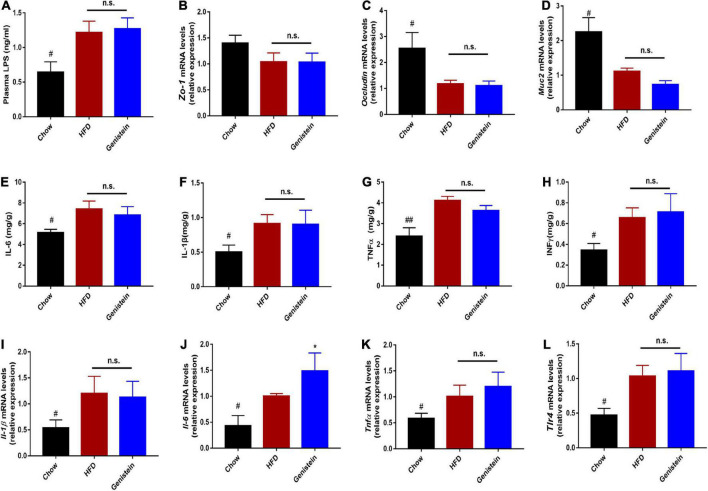
Genistein has limited effects on intestinal permeability and markers of liver inflammation **(A)** Plasma LPS level. **(B–D)** qRT-PCR mRNA analysis of *Zo-1*, *Occludin*, and *Muc2* in colon of mice. **(E–H)** Inflammatory cytokines levels in colon tissues. **(I–L)** qRT-PCR mRNA analysis of *Il-1*β, *Il-6*, *Tnf-*α and *Tlr4* in liver of mice. Data are presented as means ± SEM; *n* = 4–5 mice/group. ^#^*p* < 0.05 and ^##^*p* < 0.01 for Chow vs. HFD. *p < 0.05 for Genistein vs. HFD.

It was previously demonstrated that defects in barrier dysfunction contribute to an increased risk of obesity, diabetes, and NAFLD (21, 22). Therefore, we speculated on whether genistein had a protective effect on gut barrier. The circulating level of LPS was not altered by genistein treatment, indicating genistein treatment had limited effect on preventing metabolic endotoxemia (Figure 7A). Consistently, colonic mRNA expression of zonula occludens-1 (*Zo-1*) was no different between genistein-treated and HFD control mice (Figure 7B). Similarly, other gut barrier markers including *Occludin* and mucin2 (*Muc2*) were unaffected by genistein treatment (Figures 7C,D). Furthermore, genistein did not affect the colonic levels of cytokines (Figures 7E–H). Besides, hepatic pro-inflammatory cytokines mRNA including interlukin-6 (*Il-6*), *Il-1*β and tumor-necrosis factor (*Tnf*α) had no obvious change in genistein-treated group (Figures 7I–L). These results demonstrated that genistein has no potential in restoring intestinal barrier integrity and decreasing the levels of hepatic inflammation.

## Discussion

**FIGURE 8:**
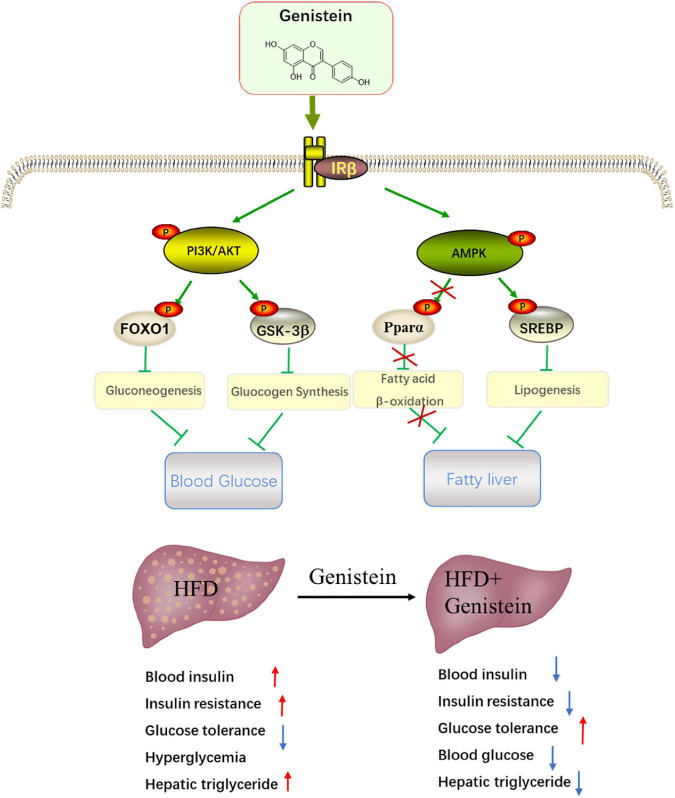
Schematic showing the potential mechanism through which genistein ameliorates hyperglycemia and fatty liver induced by HFD. Schematic diagram summarizes that genistein maintains glucose homeostasis to protect from HFD-induced insulin resistance and fatty liver by modulating insulin signaling pathway and energy status in the liver.

The liver has an essential role in maintaining glucose homeostasis. Hepatic insulin resistance is developed early in the pathophysiology of metabolic overload (23). Insulin resistance in liver will increase glucose production with stimulating hepatic lipid storage (24). Here, we reported that daily treatment of HFD-fed mice with genistein is sufficient to prevent diet-induced insulin resistance and improve glucose metabolism. Our work further elucidates the mechanism underlying the benefits of genistein treatment. The protective effect of genistein against high-fat-diet-induced hyperglycemia and fatty liver attributes to activate hepatic insulin signaling pathway (Figure 8).

Although genistein has been reported to prevent hyperglycemia in preclinical and clinical studies (16, 25, 26), the underlying mechanism responsible for genistein medicating the beneficial effects remains poorly understood. Obesity is commonly occupied with hyperglycemia. In this study, we used HFD to establish hyperglycemia mouse model. The levels of fasting blood glucose and insulin were elevated in HFD-fed mice, suggesting hyperglycemia model was established. Consistent with previous studies, our results showed that genistein ameliorated hyperglycemia and improved glucose metabolism. The OGTT experimental results showed that genistein improved the glucose tolerance. The degree of insulin resistance indicated by HOMA-IR was effectively ameliorated by genistein in HFD mice. Targeting insulin resistance may contribute to decreasing the lipid storage in liver tissue (27). Our results also suggested that genistein treatment prevented the HFD-induced hepatic lipid accumulation. Following treatment with genistein, the disturbance in liver function was attenuated, indicated by the decrease in serum ALT and AST levels.

Genistein prevented the HFD-induced decrease in hepatic insulin-signaling cascade. The liver plays a central role in the regulation of glucose metabolism (28). Insulin signaling loss in the liver results in severe insulin resistance and hepatic dysfunction (9). Insulin resistance is characterized by the reduced ability of insulin to regulate glucose homeostasis, which leads to hyperinsulinemia and metabolic disruptions (29). Activating hepatic IR and its downstream signaling molecules suppresses glucose uptake and glycogen synthesis on the liver (30). As our results suggested that reduced hyperinsulinemia and elevated HOMA-IR index were found in genistein-treated HFD mice, we hypothesized that the hepatic insulin signaling pathway might contribute to the improvement of insulin resistance by genistein administration. In this study, we found that hepatic IR signaling downstream was activated by genistein treatment, evidenced by increased phosphorylation of IRβ, PI3K, and AKT. Hence, it is speculated that the protective effect of genistein on improving insulin sensitivity is partly attributable to activating the insulin-signaling pathway.

In line with overall increased hepatic AKT activation, the phosphorylation of glycogen synthase kinase 3β (GSK3β) and FOXO1 was higher in the liver of genistein treatment. Akt phosphorylates GSK3β and FoxO1 to suppress glycogen synthesis and gluconeogenesis, respectively (31). Consistently, we found that genistein administration significantly improved fasting blood glucose, which is mainly associated with hepatic glycogenolysis and gluconeogenesis. The suppressed phosphorylation of GSK3β and FoxO1 could be the mechanism for the decrement in glucose level observed in mice treated with genistein. This hypothesis was supported by a major reduction in the level of hepatic mRNA expression of the rate-limiting enzyme involved in gluconeogenesis. FoxO1 promotes hepatic gluconeogenesis *via* increasing the expression of phosphoenolpyruvate carboxy kinase (PEPCK) and glucose-6-phosphatases (G6pc), key rate-limiting enzymes for hepatic gluconeogenesis (32). We observed lower *Pepck* and *G6pc* levels in genistein-receiver compared to HFD-receiver mice, leading to decreased gluconeogenesis in liver. Overall, these results indicated the mechanism through which genistein improved glucose metabolism in HFD-fed mice.

Insulin resistance is strongly associated with the pathogenesis of lipid accumulation in the liver (23, 33). The increased level of insulin contributes to the hepatic *de novo* lipogenesis (34). Our study provided *in vivo* and *in vitro* evidence that genistein prevented hepatic lipid deposition. Insulin signaling pathway which is associated with insulin resistance, as well as altered hepatic glycolysis and glycogen synthase, branches to alter hepatic energy status (35). Indeed, genistein markedly increased the phosphorylation of AMPK involved in hepatic lipid metabolism, which is similar to physiological stimulation altering intracellular energy status. The activation of AMPK can downregulate HFD-induced hepatic lipid storage by decreasing the protein expression of hepatic lipogenesis including SREBP1 (36). We observed that genistein decreased the protein expression of SREBP1. Our study further provided that the expression levels of ACC and FAS, two main enzymes controlled by SREBP1 (37), were suppressed by genistein treatment. AMPK activator can regulate hepatic lipid metabolism by impacting β-oxidation genes, *Ppar*α and *Pgc1*α (38, 39). As treatment of HFD mice with genistein did not have a significant effect on *Ppar*α and *Pgc1*α, this may indicate that genistein activates insulin signaling to protect liver from lipid storage without depending on β-oxidation. These results suggested that genistein balanced the lipid metabolism by regulating hepatic *de novo* but not fatty-acid oxidation.

It was of note that mRNA levels of the tight junction proteins *Zo-1* and *occluding* were significantly downregulated in proximal colon of HFD-fed mice. Pioneering research has reported that a high-fat diet can increase intestinal permeability, contributing to insulin resistance and fatty liver (40–42). Intestinal dysfunction commonly coincides with hepatic inflammation. Reducing hepatic expression of cytokines *via* improving intestinal barrier was able to prevent the progression of fatty live and insulin resistance (43). Targeting the integrity of the intestinal barrier may represent an effective strategy to prevent metabolic dysregulation (44). However, the levels of *Zo-1*, *Occluding*, and *Muc2* in proximal colon along with the mRNA levels of inflammatory cytokines in the liver were not affected at the end of genistein intervention. Despite the effects of genistein on the composition of gut microbiota being reported in previous studies (16, 45), the potential application of genistein on insulin resistance and fatty liver induced by HFD diet occurred in the lack of effect on gut permeability. Therefore, further studies will be essential to explore whether combining genistein with prebiotics has more beneficial effects.

The current study provided a novel mechanism, wherein genistein could intervene with insulin signaling pathway to improve hepatic function and glucose metabolism. Likewise, it will be important to investigate the beneficial effects of other isoflavones on glucose homeostasis and hepatic lipid-lowing properties, as well as the difference between them. The research might extend the use of genistein as a dietary supplement to combat prediabetes combined with non-alcoholic fatty liver disease. However, these outcomes should be investigated cautiously and are not sufficient to guide clinical recommendations given the limitations of the design of animal experiments. Meanwhile, the safety and efficacy of genistein in humans should be further evaluated.

## Conclusion

In conclusion, we show here that, genistein has beneficial effects on hyperglycemia and hepatic lipid accumulation in HFD-induced obesity mice through improvements in hepatic function. Genistein is capable of modulating hyperglycemia by activating the hepatic IRβ/PI3/AKT signaling pathway, increasing the phosphorylation of hepatic Foxo1/GSK3β and improving glucose metabolism of liver. The reduced level of insulin and upregulation of insulin signaling in genistein-treated mice also lead to an increase in hepatic energy status by inducing energy-sensing AMPK and inhibiting *de novo* lipogenesis to prevent hepatic lipid accumulation. These findings provide novel insights into the action mechanisms of genistein and direction for future studies addressing the use of genistein and other isoflavones in the clinical setting of prediabetes and hepatic lipid accumulation.

## Data availability statement

The original contributions presented in this study are included in the article/supplementary material, further inquiries can be directed to the corresponding author/s.

## Ethics statement

The animal study was reviewed and approved by the Animal Care and Use Committee of the School of Basic Medical Sciences, Inner Mongolia Medical University.

## Author contributions

NZ: conceptualization, investigation, writing the original draft, and funding acquisition. WZ: methodology, formal analysis, and investigation. XG and JL: visualization and validation. SL: methodology and validation. HZ: supervision and project administration. BF: supervision, project administration, and funding acquisition. All authors contributed to the article and approved the submitted version.
